# Whole genome analysis in *APOE4* homozygotes identifies the *DAB1-RELN* pathway in Alzheimer's disease pathogenesis

**DOI:** 10.1016/j.neurobiolaging.2022.07.009

**Published:** 2022-11

**Authors:** Matthew Bracher-Smith, Ganna Leonenko, Emily Baker, Karen Crawford, Andrew C. Graham, Dervis A. Salih, Brian W. Howell, John Hardy, Valentina Escott-Price

**Affiliations:** a Division of Psychological Medicine & Clinical Neurosciences, Cardiff University, Cardiff, UK; b Dementia Research Institute, Cardiff University, Cardiff, UK; c Dementia Research Institute, University College London, UK; d Neuroscience and Physiology, State University of New York, Albany, NY, USA

**Keywords:** Alzheimer's, APOE, GWAS, Epistasis

## Abstract

The *APOE*-ε4 allele is known to predispose to amyloid deposition and consequently is strongly associated with Alzheimer's disease (AD) risk. There is debate as to whether the *APOE* gene accounts for all genetic variation of the *APOE* locus. Another question which remains is whether *APOE*-ε4 carriers have other genetic factors influencing the progression of amyloid positive individuals to AD. We conducted a genome-wide association study in a sample of 5,390 *APOE*-ε4 homozygous (ε4ε4) individuals (288 cases and 5102 controls) aged 65 or over in the UK Biobank. We found no significant associations of SNPs in the *APOE* locus with AD in the sample of ε4ε4 individuals. However, we identified a novel genome-wide significant locus associated to AD, mapping to *DAB1* (rs112437613, OR = 2.28, CI = 1.73–3.01, *p* = 5.4 × 10^−9^). This identification of *DAB1* led us to investigate other components of the *DAB1-RELN* pathway for association. Analysis of the *DAB1-RELN* pathway indicated that the pathway itself was associated with AD, therefore suggesting an epistatic interaction between the *APOE* locus and the *DAB1-RELN* pathway.

## 1 Introduction

Genome-wide association studies (GWAS) have led to the identification of many genetic loci influencing the risk of dementia (Hardy and Escott-Price, 2019). However, none of these approach the importance of the *APOE* locus (Coon et al., 2007) where the *APOE-*ε4 allele has a frequency of ∼15% in controls and has a risk ratio of >3 in cases. Other loci with allele frequencies of >1% have risk ratios of <1.4. Recent studies have shown that the *APOE* genotype is almost solely responsible for amyloid deposition whereas other components of Alzheimer's disease (AD) genetic risk contribute to the occurrence of dementia in the context of amyloid deposition (Leonenko et al., 2019). Furthermore, neuropathologic studies have shown that clinical diagnoses in Alzheimer series had a diagnostic accuracy of around 80%: this accuracy is implied by analyses comparing the large clinical GWAS with the smaller neuropathologic GWAS, leading to the concern that these larger GWAS are contaminated by other diagnoses. This concern is heightened by the reports of loci for frontotemporal dementia in case series labelled as Alzheimer's disease in the most recent GWAS for the disorder (Wightman et al., 2021).

With this background, we have undertaken an AD GWAS in individuals who are *APOE-*ε4 homozygotes for 3 reasons. First, because in this group diagnostic accuracy is very high; second, to assess whether in this context there is additional genetic risk at the *APOE* locus; and third, to assess which previously reported loci are replicated in these cases and whether there are any novel loci we can identify which are dependent on *APOE-*ε4 homozygosity. This study was possible in the UK Biobank (Sudlow et al., 2015) because it has a very large cohort, with a sufficient number (for statistical analyses) of *APOE-*ε4 homozygotes, where many participants are now reaching the age where they are at risk.

Here we report that the *APOE* allele alone accounts for the AD risk in the LD block on chromosome 19 in the European population. Furthermore, in *APOE-*ε4 homozygotes, we identify AD risk associated with the *DAB1* gene that encodes a synapse regulatory protein. Subsequent analyses revealed a gene set association with the *DAB-RELN* pathway.

## 2 Material and methods

### 2.1 Phenotypes

Individuals from the UK Biobank were considered if they self-reported as white British and were of similar genetic ancestry by principal component analysis (UK Biobank field 22006), were unrelated (kinship coefficient < 0.04) and if they had not withdrawn consent to participate under UK Biobank. Participants were further excluded if they showed excessive missingness or sex chromosome aneuploidy, were outliers for heterozygosity, had mismatching self-reported and inferred sex from genotyping data, and had over 10 putative third-degree relatives. AD definition was derived using ICD-10 codes in hospital and death records. Individuals were coded as cases where dementia in Alzheimer's disease (ICD-10 code F00) or Alzheimer's disease (code G30) were present. Controls were defined as those without F00, G30, vascular dementia (F01), dementia in other diseases (F02) and unspecified dementia (F03). *APOE* status was assigned to each individual, as defined by SNPs rs7412 and rs429358 which are both present on the Affymetrix Axiom genotyping array used. After quality control and restriction to *APOE-*ε4 homozygous individuals aged 65 or over, 288 cases and 5,102 controls were included in analysis.

### 2.2 Genetic quality control

The UK Biobank genetic data from the haplotype reference consortium (HRC), imputed by the UK Biobank (Bycroft et al., 2018), was restricted to biallelic SNPs (minor allele frequency > 0.05) with Hardy-Weinberg equilibrium > 10^−6^, INFO>0.4 and posterior probability>0.4. After quality control, 5,349,830 SNPs were included in analysis.

### 2.3 Analysis

Association analysis using logistic regression was conducted in PLINK2 (Chang et al., 2015) on UK Biobank dosage data using most recently recorded age, sex and the first 15 principal components (field 22009) as covariates.

The significant findings (with the logistic regression) were further tested with Cox proportional-hazards regression (while controlling for the covariates) where the censoring occurred when a participant reported AD, allowing for the fact that some individuals have not reached the age at onset and may develop the disease given time. The code for the risk allele was the same as for the logistic regression.

The enrichment analysis of significant SNPs (at 5% significance level) or for SNPs showing the same direction of the effect (assuming that the chance to have the same direction of effect is 50%) was performed with binom.test() function in R.

The power calculations were performed with qnorm() function in *R*-statistical package at nominal 5% significance level (unless specified otherwise), where Z-score was estimated as log(OR)/var with the log(OR) as reported in the GWAS. In the Wightman et al. study (Wightman et al., 2021), the largest OR was selected from the reported ORs in the list of contributing studies. The variance estimated as the inverse variance, with allele frequencies in cases and controls (corresponding to the SNP OR), and the sample size as in our study (*N* cases = 288, *N* controls = 5,102). Plots of regional associations were created using LocusZoom (Boughton et al., 2021).

Epistasis was defined as deviation from joint 2 SNPs linear effects in the logistic regression model (known as statistical interaction). Significance of the interaction term was assessed using –epistasis option PLINK (Chang et al., 2015), accounting for the same covariates as above. The interaction plots were produced using matplotlib in python (Hunter, 2007). Following results from the GWAS, we assessed SNPs in the *DAB1* gene for epistasis. *Dab1* encodes a cytoplasmic signaling adaptor that is predominantly expressed in neurons where it acts downstream of the extracellular ligand Reelin to regulate brain lamination during development (Abadesco et al., 2014; Howell et al., 2000, 1999a; Rice et al., 1998). Since Reelin-DAB1 signaling also performs an important role in the adult brain by promoting excitatory synapse maturation (Qiu and Weeber, 2007; Ventruti et al., 2011) and modulating synaptic plasticity, learning and memory (Pujadas et al., 2010; Rogers et al., 2011; Trotter et al., 2013; Weeber et al., 2002), we also explicitly looked at the SNPs associations in the *RELN* gene (chr7:103,112,231-103,629,963).

### 2.4 DAB1-RELN pathway analysis

The Reelin ligand and DAB1 adaptor proteins are bridged by 2 partially redundant transmembrane receptors APOER2 (LRP8) and VLDLR (D'Arcangelo et al., 1999; Hiesberger et al., 1999). Reelin binding to its receptors recruits DAB1 to their cytoplasmic tails activating the SRC family kinases, SRC, FYN and YES (Arnaud et al., 2003; Bock and Herz, 2003; Hoe et al., 2006). This leads to the increased tyrosine phosphorylation of DAB1 and the recruitment of additional signaling adaptor proteins that activate 2 key branches of the pathway (Fig. 4). One branch is initiated by the binding of CRK and CRKL to phospho-DAB1, leading to the phosphorylation of C3G (*RAPGEF1*) and activation of RAP1 (*RAP1A*) (Ballif et al., 2004; Franco et al., 2011). This leads to the upregulation of N-Cadherin (*CDH2*) cell-surface expression through engagement with p120 catenin (*CTNND*) (Jossin and Cooper, 2011). A second branch is regulated by the binding of phosphatidylinostiol 3-kinase (*PIK3KA*) to DAB1 leading to the activation of PDK (*PDK1, PKD2*) and AKT (*AKT1*) ultimately suppressing the activity of the MAPT kinase GSK3 (Bock et al., 2003). In mouse, deficiency of DAB1 has been shown to augment tau-phosphorylation and Stk25 has been implicated in this process (Brich et al., 2003; Matsuki et al., 2012). Since the signaling complex and the downstream pathways have potential significance in the development of AD, we tested their associated genes for enrichment in AD.

The canonical Reelin-Dab1 signaling pathway has been studied extensively in mouse neurons and brain (Lee and D'Arcangelo, 2016). For analysis, we divided the pathway into 3 sections: (1) the receptor complex, (Reelin, the receptors ApoER2, VLDLR, the adaptor protein DAB1, and the tyrosine kinases SRC, FYN, and YES) (Arnaud et al., 2003; Bock and Herz, 2003; D'Arcangelo et al., 1999; Hiesberger et al., 1999); (2) branch 1 that regulates N-cadherin (CRK, CRKL, C3G, RAP1, P120 catenin, N-cadherin) (Ballif et al., 2004; Franco et al., 2011; Jossin and Cooper, 2011); and (3) branch 2 that is involved in microtubule-associated protein tau (MAPT) phosphorylation (PI3K, PDK, AKT, GSK3, STK25) (Bock et al., 2003; Brich et al., 2003; Matsuki et al., 2012). We converted these mouse proteins to the homologous human genes with the BioConductor function in R and the NCBI database (www.ncbi.nlm.nih.gov/) yielding: a) *RELN, VLDLR, LRP8, DAB1, SRC, FYN, YES1, b) CRK, CRKL, RAPGEF1, RAP1A, CTNND, CDH2, c) PIK3CA, PDK1, PDK2, GSK3B, AKT1, STK25, MAPT.* We tested associations in the *DAB1-RELN* pathway using individual gene-based tests, and by grouping genes into the 3 candidate pathways defined above. Gene-based analysis was run by MAGMA using FUMA v1.3.7 (Leeuw et al., 2015; Watanabe et al., 2017) using summary statistics from the GWAS. MAGMA was run using default settings; reported *p*-values are from a SNP-wise mean model. Competitive setting of MAGMA was used to test the candidate pathways for the enrichment of AD significant genes as compared to the rest of the genome.

## 3 Results

Total 288 cases and 5,102 controls were analyzed, consisting of 48.6% females in cases and 52.4% in controls, mean age 76.7 in cases and 72.9 in controls. We present the results in the following order: (1) analysis of the *APOE* locus, (2) analysis of other previously reported GWAS in these cases, (3) identification of the *DAB1* locus as a genome wide for disease, and (4) assessment of other loci in the same *DAB1-RELN* pathway.

### 3.1 *APOE* locus

No suggestive variants were identified in the *APOE* gene or surrounding region (chromosome 19: 44.5–46.5 Mb, as defined previously (Escott-Price et al., 2017)) with the lowest *p*-value at 0.003 within 1Mb of the *APOE* gene (Supplementary Fig. S1) in *APOE-*ε4 homozygotes. A logistic regression testing the effect of the *APOE* locus in all individuals (before restricting to *APOE*-ε4 homozygotes) and adjusting for age, sex and principal components, found the allelic effect of ɛ4 to be OR = 3.91 (3.65–4.18), *p* = 0 in a logistic regression on AD status. This is similar to the reported OR for the ɛ4-defining SNP in e.g. the Kunkle Stage I genome-wide association analysis (rs419358, OR = 3.33, CI = 3.20–3.45, *p* = 1.17 × 10–881) (Kunkle et al., 2019). Taking only ɛ4 homozygotes compared to ɛ3 homozygotes gives OR = 14.33 (14.30–16.61), *p* = 3.45 × 10–274. This is also consistent with previously reported estimates for ɛ4ɛ4 vs. ɛ3ɛ3 (OR = 14.49, CI = 11.91–17.64) (Genin et al., 2011).

### 3.2 Other GWAS hits

Loci previously reported as GWAS for association with Alzheimer's disease status did not show a strong replication in the current analysis of *APOE*-ε4 homozygotes only (Supplemental Table S1). Though the power to detect the GWAS-reported effect sizes in this sample is not sufficient (see last column of Supplemental Table S1), 4 loci in *CD33* (*p* = 0.004), *IQCK* (*p* = 0.009), *LILRB2* (*p* = 0.005) and *SORL1* (*p* = 0.007, MAF=0.04) had the strongest evidence for association in the current analysis and a consistent direction of effect between the current and previous GWAS. Weaker but nominally significant associations with the consistent direction of the effect were also observed in the *APH1B* (*p* = 0.024), *BIN1* (*p* = 0.011), SEC61G (*p* = 0.015) and *SNX1* (*p* = 0.048) genes. In total, 8 out of 77 SNPs (previously reported as genome-wide significant and available in our study), replicated at 0.05 significance level with the same direction of association, which is statistically greater than chance (*p* = 0.038). In addition, 53/77 (69%) SNPs have same direction of effect in the current analysis and previous GWAS which is greater than expected by chance (*p* = 0.001).

### 3.3 Identification of DAB1 as a locus

**Fig. 1:**
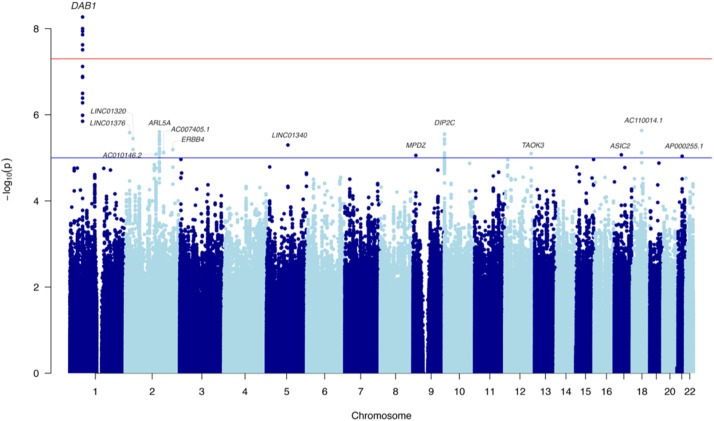
Manhattan plot for the genome-wide association study in APOE-e4 homozygotes with SNP MAF > 5%.

**Fig. 2:**
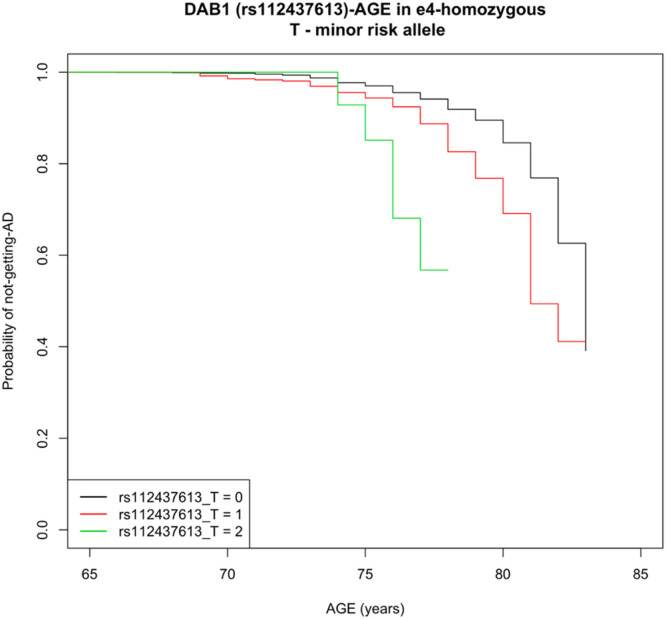
The cumulative risk of AD among APOE-e4 homozygous of the UK Biobank participants, who carry 0, 1 or 2 risk alleles T the lead SNP (rs112437613) in DAB1.

Multiple novel genome-wide significant intronic SNPs were present in *DAB1* (lead SNP: rs112437613, OR = 2.28, CI = 1.73–3.01, *p* = 5.36 × 10^−9^; Fig. 1 and Supplemental Fig. S2, Table 1). The minor allele T was associated with disease risk (MAF=6% in non-AD and 12% in AD ε4ε4-participants of the UK Biobank). To allow for the fact that some individuals might not have reached the age at onset, we fit a survival regression model (adjusting for PCs and sex). The result for the same risk allele (T) remained highly significant (Hazard Ratio=2.27, CI = 1.75–2.95, *p* = 7.8 × 10^−10^). The Kaplan–Meier graph (Fig. 2) demonstrates that probability of getting the disease (y-axis) earlier (x-axis) is higher as the number of the risk alleles of rs112437613 SNP increases.

**Table 1:** Novel genome-wide significant SNPs in DAB1.

CHR	BP	SNP	Closest gene	Current analysis	Previous LOAD GWAS (Marioni et al., 2018)						
				Effect/Alt	Freq	OR	SE	*p* value	Effect/Alt	OR	*p* value
1	57625932	rs17541203	DAB1	C/T	0.07	2.19	0.14	2.4 × 10^−8^	C/T	0.98	4.8 × 10^−1^
1	57643271	rs197111	DAB1	T/C	0.07	2.16	0.14	3.1 × 10^−8^	T/C	0.98	4.1 × 10^−1^
1	57646630	rs78921149	DAB1	T/C	0.07	2.22	0.14	1.4 × 10^−8^	T/C	0.98	4.2 × 10^−1^
1	57647715	rs112437613	DAB1	T/C	0.07	2.28	0.14	5.4 × 10^−9^	T/C	0.99	5.0 × 10^−1^
1	57648856	rs17115257	DAB1	G/T	0.08	2.12	0.13	1.0 × 10^−8^	G/T	0.99	6.5 × 10^−1^
1	57650410	rs58359668	DAB1	T/C	0.08	2.12	0.13	1.1 × 10^−8^	T/C	0.99	6.5 × 10^−1^

Key: CHR, chromosome; BP, base-pair position in build37; SNP, single nucleotide polymorphism; closest gene, genes were annotated with assembly hg19; effect/non-effect, effect and non-effect alleles; freq, frequency of reference allele in the UK Biobank *APOE*-ε4 homozygotes individuals; OR, SE, *p* value, odds ratio, standard error, and *p* value of the current and previous reported AD GWAS association studies; GWAS, reference of the corresponding GWAS study.

The frequency of this allele is reported 4%–7% in European population cohorts (1000Genomes, TOPMED, GnomAD, Estonian, ALSPAC-UK, TWINSUK, Northern Sweden, see https://genome.ucsc.edu). However, this SNP (and others in LD with it) did not show even a nominal association to AD in recent GWAS that did not preselect for the ε4ε4 genotype: e.g. a study of 21,982 cases and 41,944 controls the p-values were p∼0.5 (Table 1) (Kunkle et al., 2019).

Indeed, in a case/control sample (without screening for the *APOE*-ε4 status), the effect size of this SNP would be OR = 1.016, as the proportion of cases, with both T allele of rs112437613 and ε4ε4, is 0.016 (=MAF(ε4)^2^*MAF(rs112437613 in ε4ε4) = 0.36^2^*0.12), where 0.36 is the ε4 allele frequency in cases (Frieden and Garai, 2012), and, similarly, of controls is 0.001. Therefore, the frequencies of the T allele in the overall sample are expected to be 0.061 in cases and 0.06 in controls, and consequently, the power to detect it with the sample size of the (Kunkle et al., 2019) study is close to 0 (∼3 × 10^−7^).

**Fig. 3:**
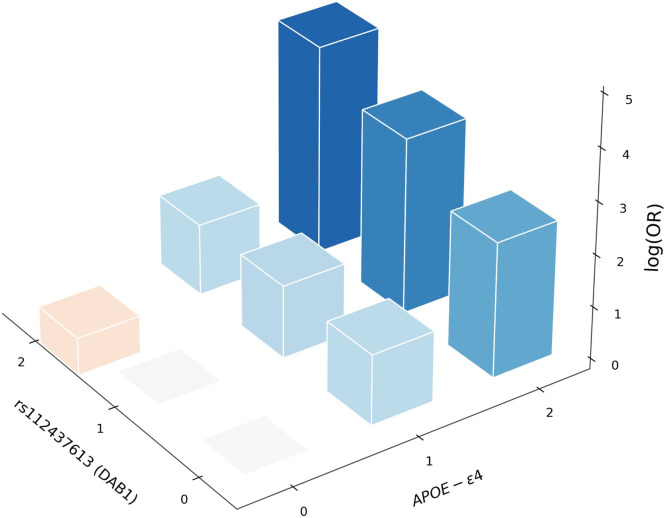
Epistatic effect between APOE-e4 and rs112437613 (DAB1) in the whole sample of the UK Biobank aged 65+ (N = 229,748). All log (odds ratio) values are with respect to the baseline homozygote with no counted alleles at both loc. Orange/red bars have negative values. All odds ratios are adjusted for age, sex and principal components.

This observation led us to test for an epistatic effect in the whole sample of the UK Biobank aged 65+ (N = 229,748). There was indeed significant epistasis between the 2 loci (interaction effect *p* = 1.5 × 10^−5^), whereas the main effect of the T allele (rs112437613) was positive (OR = 1.16, SE = 0.11), but only nominally significant (main effect *p* = 0.021), providing evidence for cooperation between these 2 loci. The risk allele frequencies in this locus depending on *APOE* and AD status are shown in Table S2 and the risk of AD, depending on the genotypes at the 2 loci, is shown in Fig. 3. The figure and table clearly show a statistical epistatic effect, where the disease risk is only visible in people with ε4ε4 genotypes.

### 3.4 Candidate analysis of other loci in the Reelin-DAB1 pathway

Following identification of an epistatic effect in *DAB1*, we assessed the lead SNP in *RELN* for statistical interaction. The *RELN* gene is comprised of 2002 SNPs and the most significantly associated SNP was rs171331137 (chr7:103479651) with OR=1.51 (SE=0.11), *p* = 2.4 × 10^−4^ (Supplemental Fig. S3). Similar to *DAB1*, we tested this SNP for interaction with *APOE*-ε4 in the whole UK Biobank sample. The interaction term was not significant (*p* = 0.24), however the pattern of AD risk based on the pair of these markers was similar to *DAB1* (Supplemental Fig. S4).

**Fig. 4:**
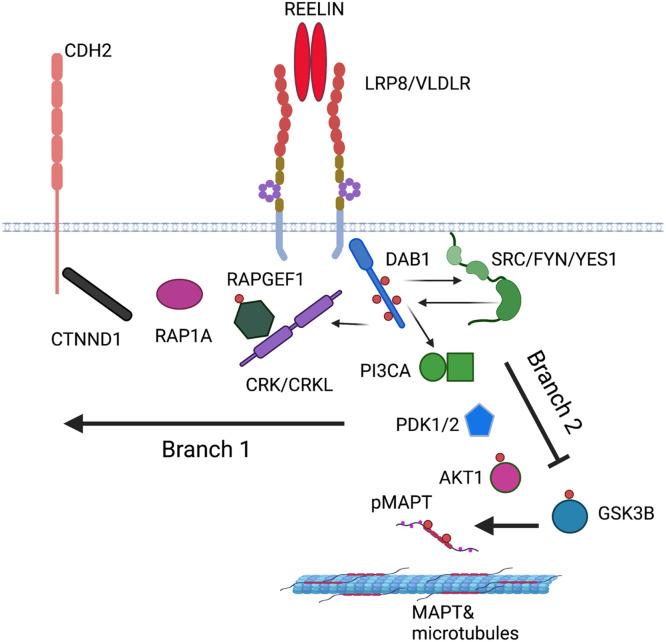
REELIN-DAB1 signaling pathway based on studies in mouse neurons and brain (human protein names are shown, created with BioRender.com, see BioRender's Academic License publication license in the Supplemental Material). The pathway branches downstream of the signaling complex. Branch 1 regulates the cell surface expression of CDH2 (N-cadherin) and branch 2 regulates MAPT phosphorylation.

We performed gene-based tests (see “DAB1-RELN pathway analysis” in Methods) on genes in the *Reelin-DAB1* pathway which highlighted nominally-significant associations in *AKT1, DAB1, PIK3CA, RELN* and *RAP1A* (Table 2). By combining genes into candidate pathways, we also tested whether the receptor complex and the 2 pathway branches contained significantly more AD associated genes as compared with the rest of the genome. We found that they were almost significantly enriched for genes associated to AD in the *APOE-*ε4 homozygotes (*p* values 0.061, 0.077, 0.083, for the receptor complex and branches 1 and 2, respectively). The strongest significance was achieved when we combined the receptor complex and the 2 branches of the pathway (*p* = 0.0061) (Fig. 4).

**Table 2:** Results of the gene-based analyses for the genes in the DAB1-RELN pathway accounting for the number of SNPs and the LD structure for each gene using MAGMA software.

*GENE*	CHR	START	STOP	N SNPs	*p* value
AKT1	14	105235686	105262080	76	0.0044
CDH2	18	25530930	25757445	471	0.164
CRK	17	1324647	1359561	90	0.660
CRKL	22	21271714	21308037	86	0.847
CTNND1	11	57520756	57586652	86	0.157
DAB1	1	57460453	58716211	3496	0.004
FYN	6	111981535	112194655	538	0.456
GSK3B	3	119540800	119813264	407	0.840
MAPT	17	43971702	44105700	669	0.397
PDK1	2	173420101	173490351	200	0.546
PDK2	17	48172101	48188733	53	0.143
PIK3CA	3	178866311	178952500	203	0.033
RELN	7	103112231	103629963	1987	0.0069
RAP1A	1	112162405	112256807	307	0.00025
RAPGEF1	9	134452157	134615364	393	0.404
SRC	20	35973088	36033835	133	0.546
VLDLR	9	2621679	2654485	103	0.158
YES1	18	721592	812327	232	0.809
LRP8	1	53708036	53793821	194	0.842
STK25	2	242434122	242449145	45	0.161

Key: GENE, gene annotation with assembly hg19; CHR- chromosome; START, STOP, start and stop base-pair positions for genes; N SNPs, the number of SNPs in the analysis, *p* value for the gene-based association test.

## 4 Discussion

### 4.1 No residual association at the APOE locus

*APOE*-ε4 is the strongest genetic risk factor for late onset AD. *APOE*-ε4 carriers have elevated risk for AD and earlier age-at-onset, with *APOE-*ε4 homozygotes at the highest risk (Corder et al., 1993; Freudenberg-Hua et al., 2018). Many loci beyond *APOE* have been reported as associated with disease in increasingly large GWAS and meta-analyses, with over 80 susceptibility loci reported collectively (Andrews et al., 2020; Bellenguez et al., 2022; Wightman et al., 2021). We find no evidence to support the role of additional loci in an extended 2Mb region around *APOE* in *APOE-*ε4 homozygotes. This is supported by previous work on risk in the *APOE* region after adjusting for number of ε4 alleles (Jun et al., 2012; Naj et al., 2011). It is therefore unlikely that variants contribute additional risk to AD in the *APOE* region in *APOE*-ε4 homozygotes although association has previously been reported in *PVRL2* and *APOC1* in Chinese samples after adjusting for number of *APOE-*ε4 alleles (Zhou et al., 2019). Variants around *APOE* may explain additional variation in risk in populations where polymorphisms are in less pronounced LD with rs429358, and residual variability in *APOE*-ε3 carriers may still modify risk for the disease (Roses et al., 2009).

### 4.2 Other established GWAS hits

This study does not have statistical power to reliably determine whether all the previously reported GWAS hits are associated with disease in *APOE-*ε4 homozygotes or whether those which do show direct evidence for association (nominal significance) are grouped in any particular pathway.

### 4.3 Association with DAB1

Putative novel risk SNPs with strong evidence for association were mapped to the *DAB1* gene on chromosome 1. Roles for *DAB1* and *RELN* have previously been suggested in AD primarily based on studies in mice (Hoe et al., 2006; Kocherhans et al., 2010; Pujadas et al., 2014; Rice et al., 2013; Rossi et al., 2020) and functional genomic analysis in humans (Gao et al., 2015), but genome-wide association in humans has been lacking. However, it has been shown that the expression of *DAB1* and *RELN* are altered in AD brains (Botella-López et al., 2006; Chin et al., 2007; Muller et al., 2011). DAB1 interacts with Asp-Pro-any residue-Tyr (NPXY) motifs in the cytoplasmic domains of amyloid precursor protein (APP) as it does with similar motifs in the cytoplasmic tails of the Reelin receptors through its N-terminal PTB domain (Howell et al., 1999b; Trommsdorff et al., 1998). The NPXY motif is required for APP internalization and its deletion reduces Aβ production (Perez et al., 1999). DAB1 association with APP has been shown to reduce amyloidogenic processing (Hoe et al., 2006), which suggests it is involved in the intracellular trafficking of APP. Reelin also reduces Aβ production in HEK293 cells that don't express DAB1 (Rice et al., 2013). In a mouse model of AD, heterozygosity of *Reln* increases the accumulation of Aβ plaques (Kocherhans et al., 2010), suggesting that the pathway physiologically alters APP cleavage in a manner that would protect against AD. In addition, homozygous loss-of-function in *Reln* and *Dab1* have been shown to augment tau-phosphorylation (Brich et al., 2003). Reelin overexpression reduces abnormal somatodendritic localization of phosphor-Tau, Aβ plaques and synaptic loss in AD model mice (Pujadas et al., 2014; Rossi et al., 2020). Thus there are links between the Reelin-DAB1 pathway and the 2 major pathological features of AD. In this study, both examined branches of the *DAB1-RELN* pathway had genes with significant association with AD. SNPs near *RAP1A* were significant; however, it remains to be determined if this branch regulates Aβ phosphor-Tau or another AD related pathology. The other major pathway downstream of Reelin-DAB1 has been associated with tau-phosphorylation and both *AKT* and *PIK3KA* from this branch were significantly associated with AD.

The dependence of the association between *DAB1*/*RELN* and AD on *APOE-*ε4 homozygosity is intriguing since there are several links between the Reelin pathway and APOE. The Reelin receptors are also APOE receptors and DAB1 binds the NPXY motifs in the cytoplasmic tails of other LDL-superfamily receptors (Howell et al., 1999b; Howell and Herz, 2001; Trommsdorff et al., 1998), such as LDL-receptor related protein 1 that has roles in APOE/Aβ internalization and clearance (Shinohara et al., 2017). Recent studies show that *APOE-*ε*4* reduces recycling of ApoER2 back to the plasma membrane making the cells less responsive to Reelin (Chen et al., 2010) and that Reelin protects against the toxic effects of Aβ on synapses (Lane-Donovan et al., 2015). Thus in *APOE*-ε4 homozygotes, one can imagine a threshold effect with high *APOE*-ε4 driving a pathological cycle by reducing the effects of DAB1 and RELN signaling including its normal function to reduce Aβ production/toxicity and/or MAPT-phosphorylation.

While the effect the SNPs have on the function of *DAB1* or other pathway genes remain to be determined, based on previous studies it would seem likely that they cause a partial loss-of-function that is potentially age dependent or cell-type specific in nature and would result in altered expression (eQTL) or splicing (sQTL). More than partial disruption of activity would likely lead to a developmental disorder in the homozygous individuals similar to loss-of-function alleles for *Dab1* in mice and *RELN* in humans and mice (Bar et al., 2003). The significant SNPs identified here fall in intron 2 and are found in 4-7% of the population. Interestingly *DAB1* exomic variation is constrained and few variants are more prevalent than 1-2% (GnomAD) suggesting that the identified SNPs do not flag an alteration in the *DAB1* coding sequence. *DAB1* is alternatively spliced and differentially expressed most notably in a cell-type specific manner (Abadesco et al., 2014; Dhananjaya et al., 2018; Gao and Godbout, 2012; Yano et al., 2010). Alternative splicing has been shown to regulate exons encoding a subset of the phosphorylation sites and a C-terminal exon altering *Dab1* functionality in mice. We note that humans have a read through variant of exon 3 that would lead to transcriptional termination 14 residues later (variant 9) that has not been identified in mice. It encodes the first part of the phosphotyrosine binding (PTB) domain residues 37–69, but it is likely to be functionally inert since the PTB domain extends to residue 171 (Howell et al., 1997). With this complexity and the size of the *DAB1* gene, over 1 Mb, it could take significant effort to dissect the consequence of the SNPs identified here on gene function and AD.

While the UK Biobank provides a large cohort and contains sufficient *APOE-*ε4 homozygous individuals for analysis, where AD status is likely to have high diagnostic accuracy, there are several limitations to the current study. First, as a prospective longitudinal cohort, participants in the UK Biobank are relatively young and at point of analysis contained fewer AD cases than are routinely observed in large case-control meta-analyses. Second, this lower sample size meant power was inadequate to replicate associations of previously-reported genome-wide significant loci for AD. Third, the UK Biobank is known to show slight difference from the general UK population (according to the last UK-wide census) with respect to characteristics such as educational attainment, socioeconomic status and gender, and these may limit general applicability of the findings to other populations (Fry et al., 2017).

In conclusion, we find a novel genome-wide significant hit in *DAB1* in an *APOE-*ε4 homozygote AD GWAS. This seems to be a hit only in *APOE-*ε4 homozygotes. Furthermore, it seems that this association marks a more general importance of the *DAB1-RELN* pathway in disease pathogenesis. It is not clear why this pathway should be of such importance in *APOE-*ε4 homozygotes only, but a clue may be that such individuals have particularly dense Aβ pathology and one can imagine that this pathway either has a role in modulating APP processing or in driving tau-phosphorylation in a manner that is dependent on high Aβ levels. This work suggests that *DAB1* has a protective role in late onset AD and highlights the importance of resolving the mechanism that likely involves the REELIN-DAB1 pathway for therapeutic development.

## Verification

The authors verify that the manuscript has not been published previously and is not under consideration for publication elsewhere and will not be published elsewhere, if accepted. Publication is approved by all authors and relevant authorities. A preprint is hosted on medarxiv (DOI:https://doi.org/10.1101/2022.04.28.22274418).

## Data availability

Data underpinning the findings in this study are available upon successful application to the UK Biobank. Derived data including GWAS summary statistics are openly available at the Cardiff University research portal, at doi:10.17035/d.2022.0216755828.

## CRediT authorship contribution statement

**Matthew Bracher-Smith:** Methodology, Formal analysis, Writing – original draft, Writing – review & editing, Visualization. **Ganna Leonenko:** Formal analysis, Investigation, Data curation. **Emily Baker:** Formal analysis, Data curation. **Karen Crawford:** Formal analysis. **Andrew C. Graham:** Data curation. **Dervis A. Salih:** Validation, Investigation, Writing – review & editing. **Brian W. Howell:** Validation, Investigation, Writing – original draft, Writing – review & editing, Visualization. **John Hardy:** Conceptualization, Validation, Investigation, Writing – original draft, Writing – review & editing, Funding acquisition. **Valentina Escott-Price:** Conceptualization, Methodology, Validation, Formal analysis, Investigation, Resources, Writing – original draft, Writing – review & editing, Visualization, Supervision, Project administration, Funding acquisition.

## Disclosure statement

The authors report no competing interests.

## Appendix Supplementary materials

Image, application 1

## References

[bib0001] Abadesco A.D., Cilluffo M., Yvone G.M., Carpenter E.M., Howell B.W., Phelps P.E. (2014). Novel disabled-1-expressing neurons identified in adult brain and spinal cord. Eur. J. Neurosci..

[bib0002] Andrews S.J., Fulton-Howard B., Goate A. (2020). Interpretation of risk loci from genome-wide association studies of Alzheimer's disease. Lancet Neurol..

[bib0003] Arnaud L., Ballif B.A., Förster E., Cooper J.A. (2003). Fyn tyrosine kinase is a critical regulator of disabled-1 during brain development. Curr. Biol..

[bib0004] Ballif B.A., Arnaud L., Arthur W.T., Guris D., Imamoto A., Cooper J.A. (2004). Activation of a Dab1/CrkL/C3G/Rap1 pathway in Reelin-stimulated neurons. Curr. Biol..

[bib0005] Bar I., Tissir F., Lambert de Rouvroit C., De Backer O., Goffinet A.M. (2003). The gene encoding disabled-1 (DAB1), the intracellular adaptor of the Reelin pathway, reveals unusual complexity in human and mouse. J. Biol. Chem..

[bib0006] Bellenguez C., Küçükali F., Jansen I.E., Kleineidam L, Moreno-Grau S., Amin N., Naj A.C., Campos-Martin R., Grenier-Boley B., Andrade V., Holmans P.A., Boland A., Damotte V., van der Lee S.J., Costa M.R., Kuulasmaa T., Yang Q., de Rojas I., Bis J.C., Yaqub A., Prokic I., Chapuis J., Ahmad S., Giedraitis V., Aarsland D., Garcia-Gonzalez P., Abdelnour C., Alarcón-Martín E., Alcolea D., Alegret M., Alvarez I., Álvarez V., Armstrong N.J., Tsolaki A., Antúnez C., Appollonio I., Arcaro M., Archetti S, Pastor A.A., Arosio B., Athanasiu L., Bailly H., Banaj N., Baquero M., Barral S., Beiser A., Pastor A.B., Below J.E., Benchek P., Benussi L., Berr C., Besse C., Bessi V., Binetti G., Bizarro A., Blesa R., Boada M., Boerwinkle E., Borroni B., Boschi S., Bossù P., Bråthen G., Bressler J., Bresner C., Brodaty H., Brookes K.J., Brusco L.I., Buiza-Rueda D., Bûrger K., Burholt V., Bush William S., Calero M., Cantwell L.B., Chene G., Chung J., Cuccaro M.L., Carracedo Á., Cecchetti R., Cervera-Carles L, Charbonnier C., Chen H.-H., Chillotti C., Ciccone S., Claassen J.A.H.R., Clark C., Conti E., Corma-Gómez A., Costantini E., Custodero C., Daian D., Dalmasso M.C., Daniele A., Dardiotis E., Dartigues J.-F., de Deyn P.P., de Paiva Lopes K., de Witte L.D., Debette S., Deckert J., del Ser T., Denning N., DeStefano A., Dichgans M., Diehl-Schmid J., Diez-Fairen M., Rossi P.D., Djurovic S., Duron E., Düzel E., Dufouil C., Eiriksdottir G., Engelborghs S., Escott-Price V., Espinosa A., Ewers M., Faber K.M., Fabrizio T., Nielsen S.F., Fardo D.W., Farotti L., Fenoglio C., Fernández-Fuertes M., Ferrari R., Ferreira C.B., Ferri E., Fin B., Fischer P., Fladby T., Fließbach K., Fongang B., Fornage M., Fortea J., Foroud T.M., Fostinelli S., Fox N.C, Franco-Macías E., Bullido M.J., Frank-García A., Froelich L., Fulton-Howard B., Galimberti D., García-Alberca J.M., García-González P., Garcia-Madrona S., Garcia-Ribas G., Ghidoni R., Giegling I., Giorgio G., Goate A.M., Goldhardt O., Gomez-Fonseca D., González-Pérez A., Graff C., Grande G., Green E., Grimmer T., Grünblatt E., Grunin M., Gudnason V., Guetta-Baranes T., Haapasalo A., Hadjigeorgiou G., Haines J.L., Hamilton-Nelson K.L., Hampel H., Hanon O., Hardy J., Hartmann A.M., Hausner L., Harwood J., Heilmann-Heimbach S., Helisalmi S., Heneka M.T., Hernández I., Herrmann M.J., Hoffmann P., Holmes C., Holstege H., Vilas R.H., Hulsman M., Humphrey J., Biessels G.J., Jian X., Johansson C., Jun G.R., Kastumata Y., Kauwe J., Kehoe P.G., Kilander L., Ståhlbom A.K., Kivipelto M., Koivisto A., Kornhuber J., Kosmidis M.H., Kukull W.A., Kuksa P.P., Kunkle B.W., Kuzma A.B., Lage C., Laukka E.J., Launer L., Lauria A., Lee C.-Y., Lehtisalo J., Lerch O., Lleó A., Longstreth W., Lopez O., de Munain A.L, Love S., Löwemark M., Luckcuck L., Lunetta K.L., Ma Y., Macías J., MacLeod C.A., Maier W., Mangialasche F., Spallazzi M., Marquié M., Marshall R., Martin E.R., Montes A.M., Rodríguez C.M., Masullo C., Mayeux R., Mead S., Mecocci P., Medina M., Meggy A., Mehrabian S., Mendoza S., Menéndez-González M., Mir P., Moebus S., Mol M., Molina-Porcel L., Montrreal L., Morelli L., Moreno F., Morgan K., Mosley T., Nöthen M.M., Muchnik C., Mukherjee S., Nacmias B., Ngandu T., Nicolas G., Nordestgaard B.G., Olaso R., Orellana A., Orsini M., Ortega G., Padovani A., Paolo C., Papenberg G, Parnetti L., Pasquier F., Pastor P., Peloso G., Pérez-Cordón A., Pérez-Tur J., Pericard P., Peters O., Pijnenburg Y.A.L., Pineda J.A., Piñol-Ripoll G., Pisanu C., Polak T., Popp J., Posthuma D., Priller J., Puerta R., Quenez O., Quintela I., Thomassen J.Q., Rábano A., Rainero I., Rajabli F., Ramakers I., Real L.M., Reinders M.J.T., Reitz C., Reyes-Dumeyer D., Ridge P., Riedel-Heller S., Riederer P., Roberto N., Rodriguez-Rodriguez E., Rongve A., Allende I.R., Rosende-Roca M., Royo J.L, Rubino E., Rujescu D., Sáez M.E., Sakka P., Saltvedt I., Sanabria Á., Sánchez-Arjona M.B., Sanchez-Garcia F., Juan P.S., Sánchez-Valle R., Sando S.B., Sarnowski C., Satizabal C.L., Scamosci M., Scarmeas N., Scarpini E., Scheltens P., Scherbaum N., Scherer M., Schmid M., Schneider A., Schott J.M., Selbæk G., Seripa D., Serrano M., Sha J., Shadrin A.A., Skrobot O., Slifer S., Snijders G.J.L., Soininen H., Solfrizzi V., Solomon A., Song Y., Sorbi S., Sotolongo-Grau O., Spalletta G., Spottke A., Squassina A., Stordal E., Tartan J.P., Tárraga L., Tesí N., Thalamuthu A., Thomas T., Tosto G., Traykov L., Tremolizzo L., Tybjærg-Hansen A., Uitterlinden A., Ullgren A., Ulstein I., Valero S., Valladares O., Broeckhoven C.Van, Vance J., Vardarajan B.N., Van der Lugt A., Dongen J.Van, Van Rooij J., van Swieten J., Vandenberghe R., Verhey F., Vidal J.-S., Vogelgsang J., Vyhnalek M., Wagner M., Wallon D., Wang L.S., Wang R., Weinhold L., Wiltfang J., Windle G., Woods B., Yannakoulia M., Zare H., Zhao Y., Zhang X., Zhu C., Zulaica M., Laczo J., Matoska V., Serpente M., Assogna F., Piras, Fabrizio, Piras, Federica, Ciullo V., Shofany J., Ferrarese C., Andreoni S., Sala G., Zoia C.P., Zompo M.Del, Benussi A., Bastiani P., Takalo M., Natunen T., Laatikainen T., Tuomilehto J., Antikainen R., Strandberg T., Lindström J., Peltonen M, Abraham R., Al-Chalabi A., Bass N.J., Brayne C., Brown K.S., Collinge J., Craig D., Deloukas, Pangiotis, Fox N., Gerrish A., Gill M., Gwilliam R., Harold D., Hollingworth P., Johnston, Jarret A., Jones L., Lawlor B., Livingston G., Lovestone S., Lupton M., Lynch A., Mann D., McGuinness B., McQuillin A., O'Donovan M.C., Owen M.J., Passmore P., Powell J.F., Proitsi P., Rossor M., Shaw C.E., Smith A.D., Gurling H., Todd S., Mummery C., Ryan N., Lacidogna G., Adarmes-Gómez A., Mauleón A., Pancho A., Gailhajenet A., Lafuente A., Macias-García D., Martín E., Pelejà E., Carrillo F., Merlín I.S., Garrote-Espina L., Vargas L., Carrion-Claro M., Marín M., Labrador M., Buendia M., Alonso M.D., Guitart M., Moreno M, Ibarria M., Periñán M., Aguilera N., Gómez-Garre P., Cañabate P., Escuela R., Pineda-Sánchez R., Vigo-Ortega R., Jesús S., Preckler S., Rodrigo-Herrero S., Diego S., Vacca A., Roveta F., Salvadori N., Chipi E., Boecker H., Laske C., Perneczky R., Anastasiou C., Janowitz D., Malik R., Anastasiou A., Parveen K., Lage C., López-García S., Antonell A., Mihova K.Y., Belezhanska D., Weber H., Kochen S., Solis P., Medel N., Lisso J., Sevillano Z., Politis D.G., Cores V., Cuesta C., Ortiz C., Bacha J.I., Rios M., Saenz A., Abalos M.S., Kohler E., Palacio D.L., Etchepareborda I., Kohler M., Novack G., Prestia F.A, Galeano P., Castaño E.M., Germani S., Toso C.R., Rojo M., Ingino C., Mangone C., Rubinsztein D.C., Teipel S., Fievet N., Deramerourt V., Forsell C., Thonberg H., Bjerke M., Roeck E.De, Martínez-Larrad M.T., Olivar N., Aguilera N., Cano A., Cañabate P., Macias J., Maroñas O., Nuñez-Llaves R., Olivé C., Pelejá E., Adarmes-Gómez A.D., Alonso M.D., Amer-Ferrer G., Antequera M., Burguera J.A., Carrillo, Fátima, Carrión-Claro M., Casajeros M.J., Martinez de Pancorbo M, Escuela, Rocío, Garrote-Espina, Lorena, Gómez-Garre, Pilar, Hevilla S., Jesús, Silvia, Espinosa M.A.L., Legaz A., López-García S., Macias-García, Daniel, Manzanares S., Marín, Marta, Marín-Muñoz J., Marín T., Martínez B., Martínez V., Martínez-Lage Álvarez P., Iriarte M.M., Periñán-Tocino M.T., Pineda-Sánchez, Rocío, Real de Asúa D., Rodrigo S., Sastre I., Vicente M.P., Vigo-Ortega, Rosario, Vivancos L., Epelbaum J., Hannequin D., campion D., Deramecourt V., Tzourio C., Brice A., Dubois B., Williams A., Thomas C., Davies C., Nash W., Dowzell K., Morales A.C., Bernardo-Harrington M., Turton J., Lord J, Brown K., Vardy E., Fisher E., Warren J.D., Rossor M., Ryan N.S., Guerreiro R., Uphill J., Bass N., Heun R., Kölsch H., Schürmann B, Lacour A., Herold C., Johnston, Janet A., Passmore P., Powell J., Patel, Yogen, Hodges A., Becker T., Warden D., Wilcock G., Clarke R., Deloukas, Panagiotis, Ben-Shlomo Y., Hooper N.M., Pickering-Brown S., Sussams R., Warner N., Bayer A., Heuser I., Drichel D., Klopp N., Mayhaus M., Riemenschneider M., Pinchler S., Feulner T., Gu W., van den Bussche H., Hüll M., Frölich L., Wichmann H.-E., Jöckel K.-H., O'Donovan M., Owen M., Bahrami S., Bosnes I., Selnes P., Bergh S., Palotie A., Daly M., Jacob H., Matakidou A., Runz H., John S., Plenge R., McCarthy M., Hunkapiller J., Ehm M., Waterworth D., Fox C., Malarstig A., Klinger K., Call, Kathy, Behrens T., Loerch P., Mäkelä T., Kaprio J., Virolainen P., Pulkki K., Kilpi T., Perola M., Frölich L., Wichmann H.-E., Jöckel K.-H., O'Donovan M., Owen M., Bahrami S., Bosnes I., Selnes P., Bergh S., Palotie A., Daly M., Jacob H., Matakidou A., Runz H., John S., Plenge R., McCarthy M., Hunkapiller J., Ehm M., Waterworth D., Fox C., Malarstig A., Klinger K., Call, Kathy, Behrens T., Loerch P., Mäkelä T., Kaprio J., Virolainen P., Pulkki K., Kilpi T., Perola M., Partanen J., Pitkäranta A., Kaarteenaho R., Vainio S., Turpeinen M., Serpi R., Laitinen T, Mäkelä J., Kosma V.-M., Kujala U., Tuovila O., Hendolin M., Pakkanen R., Waring Jeff, Riley-Gillis B., Liu J., Biswas S., Diogo D., Marshall C., Hu X., Gossel M., Graham R., Cummings B, Ripatti S., Schleutker J., Arvas M., Carpén O., Hinttala R., Kettunen J., Mannermaa A., Laukkanen J., Julkunen V., Remes A., Kälviäinen R., Peltola J., Tienari P., Rinne J., Ziemann A., Waring Jeffrey, Esmaeeli S., Smaoui N., Lehtonen A., Eaton S., Lahdenperä S., van Adelsberg J, Michon J., Kerchner G., Bowers N., Teng E., Eicher J., Mehta V., Gormley P., Linden K., Whelan C., Xu F., Pulford D., Färkkilä M., Pikkarainen S, Jussila A., Blomster T., Kiviniemi M., Voutilainen M., Georgantas B., Heap G., Rahimov F., Usiskin K., Lu T., Oh D., Kalpala K., Miller M., McCarthy L., Eklund K., Palomäki A., Isomäki P., Pirilä L., Kaipiainen-Seppänen O, Huhtakangas J., Lertratanakul A., Hochfeld M., Bing N., Gordillo J.E., Mars N., Pelkonen M., Kauppi P., Kankaanranta H., Harju T., Close D., Greenberg S., Chen, Hubert, Betts J., Ghosh S., Salomaa V., Niiranen T., Juonala M., Metsärinne K., Kähönen M., Junttila J., Laakso M., Pihlajamäki J., Sinisalo J., Taskinen M.-R., Tuomi T., Challis B., Peterson A., Chu A., Parkkinen J, Muslin A., Joensuu H., Meretoja T., Aaltonen L., Mattson J., Auranen A., Karihtala P, Kauppila S., Auvinen P., Elenius K, Popovic R., Schutzman J., Loboda A., Chhibber A., Lehtonen H., McDonough S., Crohns M., Kulkarni D., Kaarniranta K., Turunen J.A., Ollila T., Seitsonen S., Uusitalo H., Aaltonen V., Uusitalo-Järvinen H., Luodonpää M., Hautala N., Loomis S., Strauss E., Chen, Hao, Podgornaia A., Hoffman J., Tasanen K., Huilaja L., Hannula-Jouppi K., Salmi T, Peltonen S., Koulu L., Harvima I., Wu Y., Choy D., Pussinen P., Salminen A., Salo T., Rice D., Nieminen P., Palotie U., Siponen M., Suominen L., Mäntylä P., Gursoy U., Anttonen V., Sipilä K., Davis J.W., Quarless D., Petrovski S., Wigmore E., Chen C.-Y., Bronson P., Tsai E., Huang Y, Maranville J., Shaikho E., Mohammed E., Wadhawan S., Kvikstad E., Caliskan M., Chang D., Bhangale T, Pendergrass S., Holzinger E., Chen X., Hedman Å., King K.S., Wang C., Xu E., Auge F., Chatelain C., Rajpal D., Liu, Dongyu, Call, Katherine, Xia T., Brauer M., Kurki M., Karjalainen J., Havulinna A., Jalanko A., Palta P., della Briotta Parolo P., Zhou W., Lemmelä S., Rivas M, Harju J., Lehisto A., Ganna A., Llorens V., Laivuori H., Rüeger S., Niemi M.E., Tukiainen T., Reeve M.P., Heyne H., Palin K., Garcia-Tabuenca J., Siirtola H., Kiiskinen T., Lee J., Tsuo K., Elliott A., Kristiansson K., Hyvärinen K., Ritari J., Koskinen M., Pylkäs K., Kalaoja M., Karjalainen M., Mantere T., Kangasniemi E., Heikkinen S., Laakkonen E., Sipeky C., Heron S., Karlsson A., Jambulingam D., Rathinakannan V.S., Kajanne R., Aavikko M., Jiménez M.G, della Briotta Parola P., Lehistö A., Kanai M., Kaunisto M., Kilpeläinen E., Sipilä T.P., Brein G., Awaisa G., Shcherban A., Donner K., Loukola A., Laiho P., Sistonen T., Kaiharju E., Laukkanen M., Järvensivu E., Lähteenmäki S., Männikkö L., Wong R., Mattsson H., Hiekkalinna T., Paajanen T., Pärn K., Gracia-Tabuenca J., Abner E., Adams P.M., Aguirre A., Albert M.S., Albin R.L., Allen M., Alvarez L., Apostolova L.G., Arnold S.E., Asthana S., Atwood C.S., Ayres G., Baldwin C.T., Barber R.C., Barnes L.L., Barral S., Beach T.G., Becker J.T., Beecham G.W., Beekly D., Below J.E., Benchek P., Benitez B.A., Bennett D, Bertelson J., Margaret F.E., Bird T.D., Blacker D., Boeve B.F., Bowen J.D., Boxer A., Brewer J., Burke J.R., Burns J.M., Bush Will S., Buxbaum J.D., Cairns N.J., Cao C., Carlson C.S., Carlsson C.M., Carney R.M., Carrasquillo M.M., Chasse S., Chesselet M.-F., Chesi A., Chin N.A., Chui H.C., Chung J., Craft S., Crane P.K., Cribbs D.H., Crocco E.A., Cruchaga C., Cuccaro M.L., Cullum M., Darby E., Davis B., De Jager P.L., DeCarli C., DeToledo J., Dick M., Dickson D.W., Dombroski B.A., Doody R.S, Duara R., Ertekin-Taner N., Evans D.A., Fairchild T.J., Fallon K.B., Farlow M.R., Farrell J.J., Fernandez-Hernandez V., Ferris S., Frosch M.P., Fulton-Howard B., Galasko D.R., Gamboa A., Gearing M., Geschwind D.H., Ghetti B., Gilbert J.R., Grabowski T.J., Graff-Radford N.R., Grant S.F.A., Green R.C., Growdon J.H., Haines J.L., Hakonarson H., Hall J., Hamilton R.L., Harari O., Harrell L.E., Haut J., Head E., Henderson V.W., Hernandez M., Hohman T., Honig L.S., Huebinger R.M., Huentelman M.J, Hulette C.M., Hyman B.T., Hynan L.S., Ibanez L., Jarvik G.P., Jayadev S., Jin L.-W., Johnson K., Johnson L., Kamboh M.I., Karydas A.M., Katz M.J., Kaye J.A., Keene C.D., Khaleeq A., Kim R., Knebl J., Kowall N.W., Kramer J.H., Kuksa P.P., LaFerla F.M., Lah J.J., Larson E.B., Lee C.-Y., Lee E.B., Lerner A., Leung Y.Y., Leverenz J.B., Levey A.I., Li M., Lieberman A.P., Lipton R.B., Logue M., Lyketsos C.G., Malamon J., Mains D., Marson D.C., Martiniuk F., Mash D.C., Masliah E., Massman P., Masurkar A., McCormick W.C., McCurry S.M., McDavid A.N., McDonough S., McKee A.C., Mesulam M., Mez J., Miller B.L., Miller C.A., Miller J.W., Montine T.J., Monuki E.S., Morris J.C., Myers A.J., Nguyen T., O'Bryant S., Olichney J.M., Ory M., Palmer R., Parisi J.E., Paulson H.L., Pavlik V., Paydarfar D., Perez V., Peskind E., Petersen R.C., Phillips-Cremins J.E., Pierce A., Polk M., Poon W.W., Potter H., Qu L., Quiceno M., Quinn J.F., Raj A., Raskind M., Reiman E.M., Reisberg B., Reisch J.S., Ringman J.M., Roberson E.D., Rodriguear M., Rogaeva E., Rosen H.J., Rosenberg R.N., Royall D.R., Sager M.A., Sano M, Saykin A.J., Schneider J.A., Schneider L.S., Seeley W.W., Slifer S.H., Small S., Smith A.G., Smith J.P., Song Y.E., Sonnen J.A., Spina S., George-Hyslop P.S., Stern R.A., Stevens A.B., Strittmatter S.M., Sultzer D., Swerdlow R.H., Tanzi R.E., Tilson J.L., Trojanowski J.Q., Troncoso J.C., Tsuang D.W., Valladares O., Van Deerlin V.M., van Eldik L.J., Vassar R., Vinters H.V., Vonsattel J.-P., Weintraub S., Welsh-Bohmer K.A., Whitehead P.L., Wijsman E.M., Wilhelmsen K.C., Williams B, Williamson J., Wilms H., Wingo T.S., Wisniewski T., Woltjer R.L., Woon M., Wright C.B., Wu C.-K., Younkin S.G., Yu C.-E., Yu L., Zhang Y., Zhao Y., Zhu X., Adams H., Akinyemi R.O., Ali M., Armstrong N., Aparicio H.J., Bahadori M., Becker J.T., Breteler M., Chasman D., Chauhan G., Comic H., Cox S., Cupples A.L., Davies G., DeCarli C.S., Duperron M.-G., Dupuis J, Evans T., Fan F., Fitzpatrick A., Fohner A.E., Ganguli M., Geerlings M., Glatt S.J., Gonzalez H.M., Goss M., Grabe H., Habes M., Heckbert S.R., Hofer E., Hong E., Hughes T., Kautz T.F., Knol M., Kremen W., Lacaze P., Lahti J., Grand Q.Le, Litkowski E., Li S., Liu, Dan, Liu X., Loitfelder M., Manning A., Maillard P., Marioni R., Mazoyer B., van Lent D.M., Mei H., Mishra A., Nyquist P., O'Connell J., Patel, Yash, Paus T., Pausova Z., Raikkonen-Talvitie K., Riaz M., Rich S., Rotter J., Romero J, Roshchupkin G., Saba Y., Sargurupremraj M., Schmidt H., Schmidt R., Shulman J.M., Smith J., Sekhar H., Rajula R., Shin J., Simino J., Sliz E., Teumer A., Thomas A., Tin A., Tucker-Drob E., Vojinovic D., Wang Y., Weinstein G., Williams D., Wittfeld K., Yanek L., Yang Y., Farrer L.A., Psaty B.M., Ghanbari M., Raj T., Sachdev P., Mather K., Jessen F., Ikram M.A., de Mendonça A., Hort J., Tsolaki M., Pericak-Vance M.A., Amouyel P., Williams J., Frikke-Schmidt R., Clarimon J., Deleuze J.-F., Rossi G., Seshadri S., Andreassen O.A., Ingelsson M., Hiltunen M., Sleegers K., Schellenberg G.D., van Duijn C.M., Sims R., van der Flier W.M., Ruiz A., Ramirez A., Lambert J.C. (2022). New insights into the genetic etiology of Alzheimer's disease and related dementias. Nat. Genet..

[bib0007] Bock H.H., Herz J. (2003). Reelin activates Src family tyrosine kinases in neurons. Curr. Biol..

[bib0008] Bock H.H., Jossin Y., Liu P., Förster E., May P., Goffinet A.M., Herz J. (2003). Phosphatidylinositol 3-kinase interacts with the adaptor protein Dab1 in response to Reelin signaling and is required for normal cortical lamination. J. Biol. Chem..

[bib0009] Botella-López A., Burgaya F., Gavín R., García-Ayllón M.S., Gómez-Tortosa E., Peña-Casanova J., Ureña J.M., Del Río J.A., Blesa R., Soriano E., Sáez-Valero J. (2006). Reelin expression and glycosylation patterns are altered in Alzheimer's disease. Proc. Natl. Acad. Sci. U S A.

[bib0010] Boughton A.P., Welch R.P., Flickinger M., VandeHaar P., Taliun D., Abecasis G.R., Boehnke M. (2021). LocusZoom.js: interactive and embeddable visualization of genetic association study results. Bioinformatics.

[bib0011] Brich J., Shie F.S., Howell B.W., Li R., Tus K., Wakeland E.K., Jin L.W., Mumby M., Churchill G., Herz J., Cooper J.A. (2003). Genetic modulation of tau phosphorylation in the mouse. J. Neurosci..

[bib0012] Bycroft C., Freeman C., Petkova D., Band G., Elliott L.T., Sharp K., Motyer A., Vukcevic D., Delaneau O., O'Connell J., Cortes A., Welsh S., Young A., Effingham M., McVean G., Leslie S., Allen N., Donnelly P., Marchini J. (2018). The UK Biobank resource with deep phenotyping and genomic data. Nature.

[bib0013] Chang C.C., Chow C.C., Tellier L.C., Vattikuti S., Purcell S.M., Lee J.J. (2015). Second-generation PLINK: rising to the challenge of larger and richer datasets. Gigascience.

[bib0014] Chen Y., Durakoglugil M.S., Xian X., Herz J. (2010). ApoE4 reduces glutamate receptor function and synaptic plasticity by selectively impairing ApoE receptor recycling. Proc. Natl. Acad. Sci. U S A.

[bib0015] Chin J., Massaro C.M., Palop J.J., Thwin M.T., Yu G.Q., Bien-Ly N., Bender A., Mucke L. (2007). Reelin depletion in the entorhinal cortex of human amyloid precursor protein transgenic mice and humans with Alzheimer's disease. J. Neurosci..

[bib0016] Coon K.D., Myers A.J., Craig D.W., Webster J.A., Pearson J.V., Lince D.H., Zismann V.L., Beach T.G., Leung D., Bryden L., Halperin R.F., Marlowe L., Kaleem M., Walker D.G., Ravid R., Heward C.B., Rogers J., Papassotiropoulos A., Reiman E.M., Hardy J., Stephan D.A. (2007). A high-density whole-genome association study reveals that *APOE* is the major susceptibility gene for sporadic late-onset Alzheimer's disease. J. Clin. Psychiatry.

[bib0017] Corder E.H., Saunders A.M., Strittmatter W.J., Schmechel D.E., Gaskell P.C., Small G.W., Roses A.D., Haines J.L., Pericak-Vance M.A. (1993). Gene dose of apolipoprotein E type 4 allele and the risk of Alzheimer's disease in late onset families. Science.

[bib0018] Dhananjaya D., Hung K.-Y., Tarn W.-Y. (2018). RBM4 modulates radial migration via alternative splicing of Dab1 during cortex development. Mol. Cell. Biol..

[bib0019] D'Arcangelo G., Homayouni R., Keshvara L., Rice D.S., Sheldon M., Curran T. (1999). Reelin is a ligand for lipoprotein receptors. Neuron.

[bib0020] Escott-Price V., Shoai M., Pither R., Williams J., Hardy J. (2017). Polygenic score prediction captures nearly all common genetic risk for Alzheimer's disease. Neurobiol. Aging.

[bib0021] Franco S.J., Martinez-Garay I., Gil-Sanz C., Harkins-Perry S.R., Müller U. (2011). Reelin regulates cadherin function via Dab1/Rap1 to control neuronal migration and lamination in the neocortex. Neuron.

[bib0022] Freudenberg-Hua Y., Li W., Davies P. (2018). Effects of age, sex, and ethnicity on the association between apolipoprotein E genotype and Alzheimer disease: a meta-analysis. Front. Med..

[bib0023] Frieden C., Garai K. (2012). Structural differences between apoE3 and apoE4 may be useful in developing therapeutic agents for Alzheimer's disease. Proc. Natl. Acad. Sci. U S A.

[bib0024] Fry A., Littlejohns T.J., Sudlow C., Doherty N., Adamska L., Sprosen T., Collins R., Allen N.E. (2017). Comparison of sociodemographic and health-related characteristics of UK Biobank participants with those of the general population. Am. J. Epidemiol..

[bib0025] Gao H., Tao Y., He Q., Song F., Saffen D. (2015). Functional enrichment analysis of three Alzheimer's disease genome-wide association studies identities DAB1 as a novel candidate liability/protective gene. Biochem. Biophys. Res. Commun..

[bib0026] Gao Z., Godbout R. (2012). Reelin-disabled-1 signaling in neuronal migration: splicing takes the stage. Cell. Mol. Life Sci..

[bib0027] Genin E., Hannequin D., Wallon D., Sleegers K., Hiltunen M., Combarros O., Bullido M.J., Engelborghs S., de Deyn P., Berr C., Pasquier F., Dubois B., Tognoni G., Fiévet N., Brouwers N., Bettens K., Arosio B., Coto E., del Zompo M., Mateo I., Epelbaum J., Frank-Garcia A., Helisalmi S., Porcellini E., Pilotto A., Forti P., Ferri R., Scarpini E., Siciliano G., Solfrizzi V., Sorbi S., Spalletta G., Valdivieso F., Vepsäläinen S., Alvarez V., Bosco P., Mancuso M., Panza F., Nacmias B., Boss P., Hanon O., Piccardi P., Annoni G., Seripa D., Galimberti D., Licastro F., Soininen H., Dartigues J.F., Kamboh M.I., van Broeckhoven C., Lambert J.C., Amouyel P., Campion D. (2011). APOE and Alzheimer disease: a major gene with semi-dominant inheritance. Mol. Psychiatry.

[bib0028] Hardy J., Escott-Price V. (2019). Genes, pathways and risk prediction in Alzheimer's disease. Hum. Mol. Genet..

[bib0029] Hiesberger T., Trommsdorff M., Howell B.W., Goffinet A., Mumby M.C., Cooper J.A., Herz J. (1999). Direct binding of Reelin to VLDL receptor and ApoE receptor 2 induces tyrosine phosphorylation of disabled-1 and modulates tau phosphorylation. Neuron.

[bib0030] Hoe H.-S., Tran T.S., Matsuoka Y., Howell B.W., Rebeck G.W. (2006). DAB1 and Reelin effects on amyloid precursor protein and ApoE receptor 2 trafficking and processing. J. Biol. Chem..

[bib0031] Howell B.W., Gertler F.B., Cooper J.A. (1997). Mouse disabled (mDab1): a SRC binding protein implicated in neuronal development. EMBO J..

[bib0032] Howell B.W., Herrick T.M., Cooper J.A. (1999). Reelin-induced tryosine phosphorylation of disabled 1 during neuronal positioning. Genes Dev..

[bib0033] Howell B.W., Herrick T.M., Hildebrand J.D., Zhang Y., Cooper J.A. (2000). Dab1 tyrosine phosphorylation sites relay positional signals during mouse brain development. Curr. Biol..

[bib0034] Howell B.W., Herz J. (2001). The LDL receptor gene family: signaling functions during development. Curr. Opin. Neurobiol..

[bib0035] Howell B.W., Lanier L.M., Frank R., Gertler F.B., Cooper J.A. (1999). The disabled 1 phosphotyrosine-binding domain binds to the internalization signals of transmembrane glycoproteins and to phospholipids. Mol. Cell. Biol..

[bib0036] Hunter J.D. (2007). Matplotlib. Comput. Sci. Eng..

[bib0037] Jossin Y., Cooper J.A. (2011). Reelin, Rap1 and N-cadherin orient the migration of multipolar neurons in the developing neocortex. Nat. Neurosci..

[bib0038] Jun G., Vardarajan B.N., Buros J., Yu C.-E., Hawk M.V., Dombroski B.A., Crane P.K., Larson E.B., Mayeux R., Haines JL, Lunetta K.L., Pericak-Vance M.A., Schellenberg G.D., Farrer L.A. (2012). Comprehensive search for alzheimer disease susceptibility loci in the APOE region. Arch. Neurol..

[bib0039] Kocherhans S., Madhusudan A., Doehner J., Breu K.S., Nitsch R.M., Fritschy J.M., Knuesel I. (2010). Reduced Reelin expression accelerates amyloid-β plaque formation and tau pathology in transgenic Alzheimer's disease mice. J. Neurosci..

[bib0040] Kunkle B.W., Grenier-Boley B., Sims R., Bis J.C., Damotte V., Naj A.C., Boland A., Vronskaya M., Lee, van der S.J., Amlie-Wolf A., Bellenguez C., Frizatti A., Chouraki V., Martin E.R., Sleegers K., Badarinarayan N., Jakobsdottir J., Hamilton-Nelson K.L., Moreno-Grau S., Olaso R., Raybould R., Chen Y., Kuzma A.B., Hiltunen M., Morgan T., Ahmad S., Vardarajan B.N., Epelbaum J., Hoffmann P., Boada M., Beecham G.W., Garnier J.-G., Harold D., Fitzpatrick A.L., Valladares O., Moutet M.-L., Gerrish A., Smith A.v., Qu L., Bacq D., Denning N., Jian X., Zhao Y., Zompo M.del, Fox N.C., Choi S.-H., Mateo I., Hughes J.T., Adams H.H., Malamon J., Sanchez-Garcia F., Patel Y., Brody J.A., Dombroski B.A., Naranjo M.C.D., Daniilidou M., Eiriksdottir G., Mukherjee S., Wallon D., Uphill J., Aspelund T., Cantwell L.B., Garzia F., Galimberti D., Hofer E., Butkiewicz M., Fin B., Scarpini E., Sarnowski C., Bush W.S., Meslage S., Kornhuber J., White C.C., Song Y., Barber R.C., Engelborghs S., Sordon S., Voijnovic D., Adams P.M., Vandenberghe R., Mayhaus M., Cupples L.A., Albert M.S., Deyn P.P.de, Gu W., Himali J.J., Beekly D., Squassina A., Hartmann A.M., Orellana A., Blacker D., Rodriguez-Rodriguez E., Lovestone S., Garcia M.E., Doody R.S., Munoz-Fernadez C., Sussams R., Lin H., Fairchild T.J., Benito Y.A., Holmes C., Karamujić-Čomić H., Frosch M.P., Thonberg H., Maier W., Roshchupkin G., Ghetti B., Giedraitis V., Kawalia A., Li S., Huebinger R.M., Kilander L., Moebus S., Hernández I., Kamboh M.I., Brundin R., Turton J., Yang Q., Katz M.J., Concari L., Lord J., Beiser A.S., Keene C.D., Helisalmi S., Kloszewska I., Kukull W.A., Koivisto A.M., Lynch A., Tarraga L., Larson E.B., Haapasalo A., Lawlor B., Mosley T.H., Lipton R.B., Solfrizzi V., Gill M., Longstreth W.T., Montine T.J., Frisardi V., Diez-Fairen M., Rivadeneira F., Petersen R.C., Deramecourt V., Alvarez I., Salani F., Ciaramella A., Boerwinkle E., Reiman E.M., Fievet N., Rotter J.I., Reisch J.S., Hanon O., Cupidi C., Uitterlinden A.G.A., Royall D.R., Dufouil C., Maletta R.G., Rojas I.de, Sano M., Brice A., Cecchetti R., George-Hyslop P.S., Ritchie K., Tsolaki M., Tsuang D.W., Dubois B., Craig D., Wu C.-K., Soininen H., Avramidou D., Albin R.L., Fratiglioni L., Germanou A., Apostolova L.G., Keller L., Koutroumani M., Arnold S.E., Panza F., Gkatzima O., Asthana S., Hannequin D., Whitehead P., Atwood C.S., Caffarra P., Hampel H., Quintela I., Carracedo Á., Lannfelt L., Rubinsztein D.C., Barnes L.L., Pasquier F., Frölich L., Barral S., McGuinness B., Beach T.G., Johnston J.A., Becker J.T., Passmore P., Bigio E.H., Schott J.M., Bird T.D., Warren J.D., Boeve B.F., Lupton M.K., Bowen J.D., Proitsi P., Boxer A., Powell J.F., Burke J.R., Kauwe J.S.K., Burns J.M., Mancuso M., Buxbaum J.D., Bonuccelli U., Cairns N.J., McQuillin A., Cao C., Livingston G., Carlson C.S., Bass N.J., Carlsson C.M., Hardy J., Carney R.M., Bras J., Carrasquillo M.M., Guerreiro R., Allen M., Chui H.C., Fisher E., Masullo C., Crocco E.A., DeCarli C., Bisceglio G., Dick M., Ma L., Duara R., Graff-Radford N.R., Evans D.A., Hodges A., Faber K.M., Scherer M., Fallon K.B., Riemenschneider M., Fardo D.W., Heun R., Farlow M.R., Kölsch H., Ferris S., Leber M., Foroud T.M., Heuser I., Galasko D.R., Giegling I., Gearing M., Hüll M., Geschwind D.H., Gilbert J.R., Morris J., Green R.C., Mayo K., Growdon J.H., Feulner T., Hamilton R.L., Harrell L.E., Drichel D., Honig L.S., Cushion T.D., Huentelman M.J., Hollingworth P., Hulette C.M., Hyman B.T., Marshall R., Jarvik G.P., Meggy A., Abner E., Menzies G.E., Jin L.-W., Leonenko G., Real L.M., Jun G.R., Baldwin C.T., Grozeva D., Karydas A., Russo G., Kaye J.A., Kim R., Jessen F., Kowall N.W., Vellas B., Kramer J.H., Vardy E., LaFerla F.M., Jöckel K.-H., Lah J.J., Dichgans M., Leverenz J.B., Mann D., Levey A.I., Pickering-Brown S., Lieberman A.P., Klopp N., Lunetta K.L., Wichmann H.-E., Lyketsos C.G., Morgan K., Marson D.C., Brown K., Martiniuk F., Medway C., Mash D.C., Nöthen M.M., Masliah E., Hooper N.M., McCormick W.C., Daniele A., McCurry S.M., Bayer A., McDavid A.N., Gallacher J., McKee A.C., Bussche H.van den, Mesulam M., Brayne C., Miller B.L., Riedel-Heller S., Miller C.A., Miller J.W., Al-Chalabi A., Morris J.C., Shaw C.E., Myers A.J., Wiltfang J., O'Bryant S., Olichney J.M., Alvarez V., Parisi J.E., Singleton A.B., Paulson H.L., Collinge J., Perry W.R., Mead S., Peskind E., Cribbs D.H., Rossor M., Pierce A., Ryan N.S., Poon W.W., Nacmias B., Potter H., Sorbi S., Quinn J.F., Sacchinelli E., Raj A., Spalletta G., Raskind M., Caltagirone C., Bossù P., Orfei M.D., Reisberg B., Clarke R., Reitz C., Smith A.D., Ringman J.M., Warden D., Roberson E.D., Wilcock G., Rogaeva E., Bruni A.C., Rosen H.J., Gallo M., Rosenberg R.N., Ben-Shlomo Y., Sager M.A., Mecocci P., Saykin A.J., Pastor P., Cuccaro M.L., Vance J.M., Schneider J.A., Schneider L.S., Slifer S., Seeley W.W., Smith A.G., Sonnen J.A., Spina S., Stern R.A., Swerdlow R.H., Tang M., Tanzi R.E., Trojanowski J.Q., Troncoso J.C., Deerlin V.M.van, Eldik L.J.van, Vinters H.v., Vonsattel J.P., Weintraub S., Welsh-Bohmer K.A., Wilhelmsen K.C., Williamson J., Wingo T.S., Woltjer R.L., Wright C.B., Yu C.-E., Yu L., Saba Y., Pilotto A., Bullido M.J., Peters O., Crane P.K., Bennett D., Bosco P., Coto E., Boccardi V., Jager P.L.de, Lleo A., Warner N., Lopez O.L., Ingelsson M., Deloukas P., Cruchaga C., Graff C., Gwilliam R., Fornage M., Goate A.M., Sanchez-Juan P., Kehoe P.G., Amin N., Ertekin-Taner N., Berr C., Debette S., Love S., Launer L.J., Younkin S.G., Dartigues J.-F., Corcoran C., Ikram M.A., Dickson D.W., Nicolas G., Campion D., Tschanz J., Schmidt H., Hakonarson H., Clarimon J., Munger R., Schmidt R., Farrer L.A., Broeckhoven C.van, O'Donovan M.C., DeStefano A.L., Jones L., Haines J.L., Deleuze J.-F., Owen M.J., Gudnason V., Mayeux R., Escott-Price V., Psaty B.M., Ramirez A., Wang L.-S., Ruiz A., Duijn C.M.van, Holmans P.A., Seshadri S., Williams J., Amouyel P., Schellenberg G.D., Lambert J.-C., Pericak-Vance M.A. (2019). Genetic meta-analysis of diagnosed Alzheimer's disease identifies new risk loci and implicates Aβ, tau, immunity and lipid processing. Nat. Genet..

[bib0041] Lane-Donovan C., Philips G.T., Wasser C.R., Durakoglugil M.S., Masiulis I., Upadhaya A., Pohlkamp T., Coskun C., Kotti T., Steller L., Hammer R.E., Frotscher M., Bock H.H., Herz J. (2015). Reelin protects against amyloid β toxicity in vivo. Sci. Signal.

[bib0042] Lee G.H., D'Arcangelo G. (2016). New insights into reelin-mediated signaling pathways. Front. Cell. Neurosci..

[bib0043] Leeuw C.A.de, Mooij J.M., Heskes T., Posthuma D. (2015). MAGMA: generalized gene-set analysis of GWAS data. PLoS Comput. Biol..

[bib0044] Leonenko G., Shoai M., Bellou E., Sims R., Williams J., Hardy J., Escott-Price V. (2019). Genetic risk for Alzheimer disease is distinct from genetic risk for amyloid deposition. Ann. Neurol..

[bib0045] Marioni R.E., Harris S.E., Zhang Q., McRae A.F., Hagenaars S.P., Hill W.D., Davies G., Ritchie C.W., Gale C.R., Starr J.M., Goate A.M., Porteous D.J., Yang J., Evans K.L., Deary I.J., Wray N.R., Visscher P.M. (2018). GWAS on family history of Alzheimer's disease. Transl. Psychiatry.

[bib0046] Matsuki T., Zaka M., Guerreiro R., van der Brug M.P., Cooper J.A., Cookson M.R., Hardy J.A., Howell B.W. (2012). Identification of Stk25 as a genetic modifier of tau phosphorylation in Dab1-mutant mice. PLoS One.

[bib0047] Muller T., Loosse C., Schrotter A., Schnabel A., Helling S., Egensperger R., Marcus K. (2011). The AICD interacting protein DAB1 is up-regulated in Alzheimer frontal cortex brain samples and causes deregulation of proteins involved in gene expression changes. Curr. Alzheimer Res..

[bib0048] Naj A.C., Jun G., Beecham G.W., Wang L.-S., Vardarajan B.N., Buros J., Gallins P.J., Buxbaum J.D., Jarvik G.P., Crane P.K., Larson E.B., Bird T.D., Boeve B.F., Graff-Radford N.R., De Jager P.L., Evans D., Schneider J.A., Carrasquillo M.M., Ertekin-Taner N., Younkin S.G., Cruchaga C., Kauwe J.S.K., Nowotny P., Kramer P., Hardy J., Huentelman M.J., Myers A.J., Barmada M.M., Demirci F.Y., Baldwin C.T., Green R.C., Rogaeva E., George-Hyslop P.S., Arnold S.E., Barber R., Beach T., Bigio E.H., Bowen J.D., Boxer A., Burke J.R., Cairns N.J., Carlson C.S., Carney R.M., Carroll S.L., Chui H.C., Clark D.G., Corneveaux J., Cotman C.W., Cummings J.L., DeCarli C., DeKosky S.T., Diaz-Arrastia R., Dick M., Dickson D.W., Ellis W.G., Faber K.M., Fallon K.B., Farlow M.R., Ferris S., Frosch M.P., Galasko D.R., Ganguli M., Gearing M., Geschwind D.H., Ghetti B., Gilbert J.R., Gilman S., Giordani B., Glass J.D., Growdon J.H., Hamilton R.L., Harrell L.E., Head E., Honig L.S., Hulette C.M., Hyman B.T., Jicha G.A., Jin L.-W., Johnson N., Karlawish J., Karydas A., Kaye J.A., Kim R., Koo E.H., Kowall N.W., Lah J.J., Levey A.I., Lieberman A.P., Lopez O.L., Mack W.J., Marson D.C., Martiniuk F., Mash D.C., Masliah E., McCormick W.C., McCurry S.M., McDavid A.N., McKee A.C., Mesulam M., Miller B.L., Miller C.A., Miller J.W., Parisi J.E., Perl D.P., Peskind E., Petersen R.C., Poon W.W., Quinn J.F., Rajbhandary R.A., Raskind M., Reisberg B., Ringman J.M., Roberson E.D., Rosenberg R.N., Sano M., Schneider L.S., Seeley W., Shelanski M.L., Slifer M.A., Smith C.D., Sonnen J.A., Spina S., Stern R.A., Tanzi R.E., Trojanowski J.Q., Troncoso J.C., Van Deerlin V.M., Vinters H.V, Vonsattel J.P., Weintraub S., Welsh-Bohmer K.A., Williamson J., Woltjer R.L., Cantwell L.B., Dombroski B.A., Beekly D., Lunetta K.L., Martin E.R., Kamboh M.I., Saykin A.J., Reiman E.M., Bennett D.A., Morris J.C., Montine T.J., Goate A.M., Blacker D., Tsuang D.W., Hakonarson H., Kukull W.A., Foroud T.M., Haines J.L., Mayeux R., Pericak-Vance M.A., Farrer L.A., Schellenberg G.D. (2011). Common variants at MS4A4/MS4A6E, CD2AP, CD33 and EPHA1 are associated with late-onset Alzheimer's disease. Nat. Genet..

[bib0049] Perez R.G., Soriano S., Hayes J.D., Ostaszewski B., Xia W., Selkoe D.J., Chen X., Stokin G.B., Koo E.H. (1999). Mutagenesis identifies new signals for β-amyloid precursor protein endocytosis, turnover, and the generation of secreted fragments, including Aβ42. J. Biol. Chem..

[bib0050] Pujadas L., Gruart A., Bosch C., Delgado L., Teixeira C.M., Rossi D., De Lecea L., Martínez A., Delgado-García J.M., Soriano E. (2010). Reelin regulates postnatal neurogenesis and enhances spine hypertrophy and long-term potentiation. J. Neurosci..

[bib0051] Pujadas L., Rossi D., Andrés R., Teixeira C.M., Serra-Vidal B., Parcerisas A., Maldonado R., Giralt E., Carulla N., Soriano E. (2014). Reelin delays amyloid-beta fibril formation and rescues cognitive deficits in a model of Alzheimer's disease. Nat. Commun..

[bib0052] Qiu S., Weeber E.J. (2007). Reelin signaling facilitates maturation of CA1 glutamatergic synapses. J. Neurophysiol..

[bib0053] Rice D.S., Sheldon M., D'Arcangelo G., Nakajima K., Goldowitz D., Curran T. (1998). Disabled-1 acts downstream of Reelin in a signaling pathway that controls laminar organization in the mammalian brain. Development.

[bib0054] Rice H.C., Young-Pearse T.L., Selkoe D.J. (2013). Systematic evaluation of candidate ligands regulating ectodomain shedding of Amyloid precursor protein. Biochemistry.

[bib0055] Rogers J.T., Rusiana I., Trotter J., Zhao L., Donaldson E., Pak D.T.S., Babus L.W., Peters M., Banko J.L., Chavis P., Rebeck G.W., Hoe H.S., Weeber E.J. (2011). Reelin supplementation enhances cognitive ability, synaptic plasticity, and dendritic spine density. Learn. Memory.

[bib0056] Roses A.D., Lutz M.W., Amrine-Madsen H., Saunders A.M., Crenshaw D.G., Sundseth S.S., Huentelman M.J., Welsh-Bohmer K.A., Reiman E.M. (2009). A TOMM40 variable-length polymorphism predicts the age of late-onset Alzheimer's disease. Pharmacogenom. J..

[bib0057] Rossi D., Gruart A., Contreras-Murillo G., Muhaisen A., Ávila J., Delgado-García J.M., Pujadas L., Soriano E. (2020). Reelin reverts biochemical, physiological and cognitive alterations in mouse models of tauopathy. Prog. Neurobiol..

[bib0058] Shinohara M., Tachibana M., Kanekiyo T., Bu G. (2017). Role of LRP1 in the pathogenesis of Alzheimer's disease: evidence from clinical and preclinical studies: thematic review series: ApoE and lipid homeostasis in Alzheimer's disease. J. Lipid Res..

[bib0059] Sudlow C., Gallacher J., Allen N., Beral V., Burton P., Danesh J., Downey P., Elliott P., Green J., Landray M., Liu B., Matthews P., Ong G., Pell J., Silman A., Young A., Sprosen T., Peakman T., Collins R. (2015). UK Biobank: an open access resource for identifying the causes of a wide range of complex diseases of middle and old age. PLoS Med..

[bib0060] Trommsdorff M., Borg J.P., Margolis B., Herz J. (1998). Interaction of cytosolic adaptor proteins with neuronal apolipoprotein E receptors and the amyloid precursor protein. J. Biol. Chem..

[bib0061] Trotter J., Lee G.H., Kazdoba T.M., Crowell B., Domogauer J., Mahoney H.M., Franco S.J., Müller U., Weeber E.J., D'Arcangelo G. (2013). Dab1 is required for synaptic plasticity and associative learning. J. Neurosci..

[bib0062] Ventruti A., Kazdoba T.M., Niu S., D'Arcangelo G. (2011). Reelin deficiency causes specific defects in the molecular composition of the synapses in the adult brain. Neuroscience.

[bib0063] Watanabe K., Taskesen E., van Bochoven A., Posthuma D. (2017). Functional mapping and annotation of genetic associations with FUMA. Nat. Commun..

[bib0064] Weeber E.J., Beffert U., Jones C., Christian J.M., Förster E., David Sweatt J., Herz J. (2002). Reelin and ApoE receptors cooperate to enhance hippocampal synaptic plasticity and learning. J. Biol. Chem..

[bib0065] Wightman D.P., Jansen I.E., Savage J.E., Shadrin A.A., Bahrami S., Holland D., Rongve A., Børte S., Winsvold B.S., Drange O.K., Martinsen A.E., Skogholt A.H., Willer C., Bråthen G., Bosnes I., Nielsen J.B., Fritsche L.G., Thomas L.F., Pedersen L.M., Gabrielsen M.E., Johnsen M.B., Meisingset T.W., Zhou W., Proitsi P., Hodges A., Dobson R., Velayudhan L., Agee M., Aslibekyan S., Babalola E., Bell R.K., Bielenberg J., Bryc K., Bullis E., Cameron B., Coker D., Partida G.C., Dhamija D., Das S., Elson S.L., Filshtein T., Fletez-Brant K., Fontanillas P., Freyman W., Gandhi P.M., Hicks B., Hinds D.A., Huber K.E., Jewett E.M., Jiang Y., Kleinman A., Kukar K., Lane V., Lin K.H., Lowe M., Luff M.K., McCreight J.C., McIntyre M.H., McManus K.F., Micheletti S.J., Moreno M.E., Mountain J.L., Mozaffari S.V., Nandakumar P., Noblin E.S., O'Connell J., Petrakovitz A.A., Poznik G.D., Schumacher M., Shastri A.J., Shelton J.F., Shi J., Shringarpure S., Tian C., Tran V., Tung J.Y., Wang X., Wang W., Weldon C.H., Wilton P., Sealock J.M., Davis L.K., Pedersen N.L., Reynolds C.A., Karlsson I.K., Magnusson S., Stefansson H., Thordardottir S., Jonsson P.V., Snaedal J., Zettergren A., Skoog I., Kern S., Waern M., Zetterberg H., Blennow K., Stordal E., Hveem K., Zwart J.A., Athanasiu L., Selnes P., Saltvedt I., Sando S.B., Ulstein I., Djurovic S., Fladby T., Aarsland D., Selbæk G., Ripke S., Stefansson K., Andreassen O.A., Posthuma D. (2021). A genome-wide association study with 1,126,563 individuals identifies new risk loci for Alzheimer's disease. Nat. Genet..

[bib0066] Yano M., Hayakawa-Yano Y., Mele A., Darnell R.B. (2010). Nova2 regulates neuronal migration through an RNA switch in disabled-1 signaling. Neuron.

[bib0067] Zhou X., Chen Yu, Mok K.Y., Kwok T.C.Y., Mok V.C.T., Guo Q., Ip F.C., Chen Y., Mullapudi N., Giusti-Rodríguez P., Sullivan P.F., Hardy J., Fu A.K.Y., Li Y., Ip N.Y. (2019). Non-coding variability at the APOE locus contributes to the Alzheimer's risk. Nat. Commun..

